# Supplementary material for: Reconstructing the demographic history of divergence between European river and brook lampreys using approximate Bayesian computations
Source: PeerJ. 2016 Apr 7;4:e1910. doi: 10.7717/peerj.1910 (PMC4830234; doi:10.7717/peerj.1910)
Supplement: Supplemental Information 2 [file peerj-04-1910-s002.pdf]

Table S1: **Pairwise F_{ST} values**. Non-significant values are in italic and grey-colored.

	LF Aa	LF Hem	LF Beth	LF Bre	LF Ris	LF Oir	LP Aa	LP Hem	LP Bet	LP Bre	LP Ris
LF Hem	*0.000*										
LF Beth	*0.002*	*0.001*									
LF Bre	*0.000*	*0.000*	*0.003*								
LF Ris	*0.000*	*0.000*	*0.001*	*0.003*							
LF Oir	*0.000*	*0.003*	*0.011*	*0.006*	0.003						
LP Aa	0.084	0.090	0.079	0.090	0.088	0.090					
LP Hem	0.079	0.077	0.088	0.083	0.071	0.066	0.102				
LP Bet	*0.022*	*0.025*	*0.028*	0.027	0.032	*0.020*	0.115	0.090			
LP Bre	0.099	0.116	0.135	0.091	0.102	0.087	0.192	0.189	0.088		
LP Ris	0.033	0.035	0.050	0.034	0.033	0.030	0.121	0.103	0.055	0.097	
LP Oir	0.040	0.048	0.044	0.044	0.046	0.028	0.075	0.087	0.040	0.087	0.063

Table S2: **Confidence measure in model selection** inferred from 1000 pseudo observed dataset

River	Focal scenario	Type I error	Type II error				
			SI	IM	AM	SC	PAN
**AA**	I	0.435	-	0.071	0.478	0.077	0.194
	IM	0.404	0.048	-	0.078	0.685	0.427
	AM	0.522	0.352	0.071	-	0.049	0
	SC	0.811	0.035	0.071	0.051	-	0.049
	PAN	1	0	0.071	0	0	-
**BET**	I	0.989	-	0.007	0.012	0.005	0
	IM	0.326	0.027	-	0.049	0.763	0.975
	AM	0.988	0.573	0.007	-	0.106	0
	SC	0.897	0.387	0.007	0.298	-	0.106
	PAN	0.975	0.002	0.007	0	0.023	-
**BRE**	I	0.73	-	0.098	0.109	0.087	0
	IM	0.698	0.086	-	0.186	0.253	0.015
	AM	0.891	0.107	0.098	-	0.058	0
	SC	0.4	0.537	0.098	0.614	-	0.058
	PAN	0.741	0	0.098	0	0.002	-
**HEM**	I	0.632	-	0.107	0.17	0.076	0
	IM	0.744	0.151	-	0.354	0.337	0.941
	AM	0.83	0.37	0.107	-	0.051	0
	SC	0.464	0.111	0.107	0.294	-	0.051
	PAN	1	0	0.107	0	0	-
**OIR**	I	0.885	-	0.02	0.058	0.003	0
	IM	0.742	0.236	-	0.263	0.226	0.062
	AM	0.942	0.491	0.02	-	0.0119	0
	SC	0.925	0.119	0.02	0.169	-	0.019
	PAN	0.16	0.039	0.02	0.04	0.677	-
**RIS**	I	0.876	-	0.041	0.115	0.051	0
	IM	0.868	0.2	-	0.328	0.151	0.01
	AM	0.885	0.647	0.041	-	0.026	0
	SC	0.698	0.029	0.041	0.095	-	0.026
	PAN	0.011	0	0.016	0.041	0.047	-

Table S3: Confidence measure in model selection inferred from 1000 pseudo observed dataset by pairs for the strict isolation (SI) model and panmictic (PAN) model.

		Type I error						Type II error					
**Focal model**		**AA**	**BET**	**BRE**	**HEM**	**OIR**	**RIS**	**AA**	**BET**	**BRE**	**HEM**	**OIR**	**RIS**
SI versus	IM	0	0	0	0	0	0	0	0.092	0.031	0.087	0.037	0.066
	AM	0.337	0.949	0.078	0.283	0.745	0.98	0.609	0.103	0.881	0.7	0.214	0.046
	SC	0	0	0	0	0	0	0	0.068	0.029	0.073	0.023	0.05
	PAN	0	0.001	0	0	0.01	0.004	0	0	0	0	0.106	0

		Type I error						Type II error					
**Focal model**		**AA**	**BET**	**BRE**	**HEM**	**OIR**	**RIS**	**AA**	**BET**	**BRE**	**HEM**	**OIR**	**RIS**
PAN versus	**IM**	0	0	0	0.002	0	0	0.005	0.023	0	0	0.009	0.027
	**AM**	0	0	0	0.001	0	0	0.001	0.01	0	0	0.095	0.022
	**SC**	0	0	0	0	0	0	0	0	0	0	0	0
	**SI**	0	0	0	0	0	0	0	0.001	0	0	0.01	0.004

Table S4: **Estimation of variable importance from random-forest analyses**
Estimation is performed pairwise for computational tractability.
For each pairwise comparison, the three most discriminatory variables are in bold.

	MODEL											
Summary Stat	IvsAM	IvsIM	IvsSC	IvsPAN	AmvsIM	Scvs AM	AmvsPAN	SCvsIM	ImvsPAN	PANvsIM	ScvsPAN	Average
mean He pop1*	**76.64**	31.58	30.98	18.37	35.76	38.31	19.66	24.23	37.28	37.28	38.94	32.62
var He pop1	55.06	26.87	25.24	12.90	24.56	27.62	10.53	19.00	27.76	27.76	27.45	23.35
mean He pop2*	45.32	27.99	29.70	19.03	38.88	40.61	21.76	18.55	46.75	46.75	48.50	37.16
var He pop2	37.46	21.60	22.84	13.88	33.64	35.13	13.10	17.17	29.06	29.06	28.48	25.33
mean He total	58.42	**48.69**	**48.79**	17.88	**67.61**	**66.55**	24.14	25.90	42.59	42.59	45.80	41.26
var He total	42.27	35.04	32.25	16.30	32.66	36.05	12.12	20.94	28.28	28.28	28.18	25.64
mean Ar pop1	**90.18**	27.56	28.04	13.33	35.17	36.22	13.05	25.68	30.79	30.79	26.85	27.23
var Ar pop1	49.78	23.27	22.09	10.40	36.39	38.03	14.59	19.10	25.36	25.36	25.48	24.66
mean Ar pop2	57.54	25.95	25.37	13.16	34.15	31.60	15.00	28.02	22.31	22.31	22.68	23.65
var Ar pop2	46.81	25.68	22.56	10.06	36.83	35.89	8.23	23.40	16.03	16.03	18.05	19.61
mean Ar total	56.41	38.12	44.20	14.32	43.63	52.08	16.48	23.71	27.01	27.01	24.98	28.54
var Ar total	61.87	21.91	22.81	11.07	31.53	35.19	11.97	19.85	23.42	23.42	25.35	23.20
mean R pop1	47.75	30.25	30.34	8.61	37.93	39.47	10.77	21.22	21.85	21.85	20.81	22.66
var R pop1	57.89	41.89	42.03	7.08	43.97	47.00	9.64	27.19	18.67	18.67	18.13	23.22
mean R pop2	59.87	27.06	32.17	11.53	26.88	32.14	12.87	27.23	26.38	26.38	23.35	24.72
var R pop2	69.44	36.76	46.21	9.39	39.87	46.10	9.37	23.71	21.94	21.94	18.44	23.58
mean R total	44.62	27.08	28.98	11.01	32.01	35.34	11.42	17.09	22.67	22.67	22.07	21.88
var R total	46.63	21.70	21.47	7.86	30.13	29.35	9.54	21.86	19.34	19.34	16.84	19.38
var GW pop1	**94.41**	39.08	43.83	11.70	37.76	37.70	11.88	22.05	28.71	28.71	28.28	26.22
mean GW pop1	50.64	25.46	20.65	9.07	37.06	38.73	6.98	12.90	13.26	13.26	16.00	16.85
var GW pop2	64.23	31.60	37.27	12.00	31.62	37.62	12.35	25.52	26.56	26.56	25.91	25.75
mean GW pop2	43.42	26.60	21.40	7.83	40.62	38.83	7.77	8.55	15.02	15.02	14.02	16.54
var GW total	59.44	31.61	31.51	9.88	35.88	38.19	10.89	20.88	26.82	26.82	27.07	25.11
mean GW total	39.53	24.50	22.06	6.14	38.91	34.47	5.46	18.77	12.46	12.46	13.00	16.10
mean GST	65.74	**62.09**	**66.67**	**34.69**	**80.61**	**85.93**	**57.28**	**34.11**	**84.59**	**84.59**	**84.41**	**71.82**
var GST	37.25	37.52	38.70	27.29	51.50	50.54	40.93	**30.75**	**104.78**	**104.78**	**107.81**	**73.27**
mean deltamu^2	71.55	**74.59**	**72.38**	**52.09**	**76.51**	**76.05**	**61.36**	**33.14**	**103.26**	**103.26**	**98.59**	**79.28**
var deltamu^2	60.48	43.46	44.62	**41.85**	47.22	48.70	**45.17**	23.59	57.10	57.10	60.18	48.64

*Pop 1 = *Lampetra fluviatilis* population
*Pop 2 = *Lampetra planeri* population

Table S5: **Robustness of summary statistics computation under the two best models (IM and SC).** Estimation based on 10 00 dataset drawn from the posterior distribution of the parameters. Summary statistics that deviate significantly from the observed distribution are in bold.

	**IM**					**SC**					**PAN**
Statistics	AA	BET	BRE	HEM	RIS	AA	BET	BRE	HEM	RIS	OIR
mean_He_pop1	0.9915	0.9912	0.997	0.9888	1	1	1	1	0.9485	0.9994	1
**var_He_pop1**	**4.00E-004**	**2.00E-004**	**0.002**	**4.00E-004**	**0**	**0**	**1.00E-004**	**0**	**0.0089**	**0**	1
mean_He_pop2	0.8836	0.9962	0.962	0.8489	1	0.948	1	1	0.8033	0.9859	1
**var_He_pop2**	**0.0471**	**1.00E-004**	**0.01**	**0.0156**	**6.00E-004**	**0.033**	**0**	**0**	**0.0451**	**0.0316**	1
mean_He_total	0.9539	0.9984	0.999	0.9752	1	0.997	1	1	0.9111	0.9995	1
**var_He_total**	**0.0011**	**0**	**0.002**	**1.00E-004**	**0**	**0.01**	**0**	**0**	**0.011**	**0**	1
mean_A_pop1	0.9909	0.9912	0.999	0.9889	1	1	1	1	0.9491	0.9994	1
**var_A_pop1**	**4.00E-004**	**5.00E-004**	**0.001**	**4.00E-004**	**0**	**0**	**1.00E-004**	**0**	**0.0097**	**0**	1
mean_A_pop2	0.887	0.9962	0.967	0.8542	1	0.962	1	1	0.8079	0.9864	1
**var_A_pop2**	**0.0384**	**1.00E-004**	**0.01**	**0.0132**	**7.00E-004**	**0.031**	**0**	**0**	**0.0405**	**0.0342**	1
mean_A_total	0.9549	0.9984	0.999	0.9765	1	0.997	1	1	0.9128	0.9995	1
**var_A_total**	**0.0011**	**0**	**0.002**	**0**	**0**	**0.01**	**0**	**0**	**0.0103**	**0**	1
mean_Ar_pop1	0.9928	0.9906	1	0.9978	1	1	1	1	0.9776	0.9999	1
var_Ar_pop1	0.2832	0.3585	0.435	0.2961	0.4841	0.598	0.4826	0.732	0.2514	0.6358	**0**
mean_Ar_pop2	0.9075	0.9962	0.934	0.9931	1	0.999	1	1	0.8948	0.99	1
var_Ar_pop2	0.6802	0.4688	**0.045**	0.3868	0.5767	0.882	0.4959	0.352	0.4541	0.6036	**0**
mean_Ar_total	0.9909	0.9999	0.98	0.9982	1	1	1	0.999	0.9849	0.9991	1
var_Ar_total	0.3833	0.4709	0.109	0.4236	0.6422	0.629	0.569	0.376	0.3807	0.6889	**0**
mean_V_pop1	0.9926	0.9906	1	0.9978	1	1	1	1	0.9767	0.9999	1
var_V_pop1	0.2701	0.3257	0.439	0.2796	0.4769	0.586	0.4457	0.736	0.2421	0.6298	0
mean_V_pop2	0.9057	0.996	0.928	0.9924	1	0.999	1	1	0.8929	0.9895	1
var_V_pop2	0.6692	0.4395	**0.044**	0.3845	0.5762	0.874	0.458	0.341	0.4504	0.6036	**0**
mean_V_total	0.9909	0.9999	0.979	0.9981	1	1	1	0.999	0.9845	0.9987	1
var_V_total	0.3747	0.4431	0.109	0.4195	0.638	0.621	0.5385	0.377	0.3764	0.6837	**0**
mean_R_pop1	0.9785	0.9916	1	0.9916	1	0.999	1	1	0.9508	0.9999	1
var_R_pop1	0.8185	0.9522	0.989	0.8252	0.9995	0.916	0.9818	1	0.7275	0.9991	1
mean_R_pop2	0.974	0.9997	0.164	0.9702	0.9999	1	1	0.713	0.8687	0.9832	1
var_R_pop2	0.9956	0.9867	**0.006**	0.6688	0.962	1	0.9954	0.249	0.5827	0.9346	1
mean_R_total	0.9874	1	0.441	0.9884	1	1	1	0.848	0.9425	0.9988	**0**
var_R_total	0.8553	0.9729	0.108	0.8635	0.9782	0.934	0.9892	0.635	0.787	0.967	**0**
mean_GW_pop1	0.4724	**0.0458**	0.075	0.5732	**4.00E-004**	0.513	**0.0192**	**0.009**	0.6205	**0.003**	**0**
var_GW_pop1	0.783	0.6298	0.954	0.8314	0.6711	0.83	0.6003	0.921	0.8376	0.7077	**0**
mean_GW_pop2	**0.0285**	**0.0242**	0.99	**0.0197**	0.2775	**0.002**	**0.0055**	0.901	0.0849	0.2945	**0**
var_GW_pop2	0.9567	0.5846	0.182	**0**	0.462	0.967	0.5361	0.216	**0.012**	0.4693	1
mean_GW_total	0.4408	**0.0326**	1	0.7969	0.0809	0.456	**0.0119**	0.975	0.8305	0.0972	1
var_GW_total	0.7364	0.6681	0.907	0.9359	0.2853	0.794	0.6458	0.852	0.9416	0.2726	**0**
mean_GST	0.2031	0.3586	0.546	0.5067	0.7748	0.191	0.2718	0.015	0.3924	0.6114	**0**
**var_GST**	0.0845	**0.0245**	0.76	0.2878	0.2154	0.046	**0.0045**	**0.091**	0.3036	0.2239	1
mean_deltamu2	0.9328	0.9917	0.117	0.8329	0.979	0.976	0.9969	0.181	0.7456	0.9464	1
var_deltamu2	0.9387	0.9883	0.17	0.6931	0.9705	0.979	0.9944	0.243	0.6096	0.9378	1

Table S6: Untransformed Estimates of demographic parameters under the model of ongoing migration (IM) and secondary contact (SC) in each river. Θ=4*Nref*µ with µ the mutation rate. Lf = *lampetra fluviatilis*, Lp = *Lampetra planeri*. * the PAN model is controlled by a single parameter the effective population size of the single population (made of both Lf and Lp backgrounds).

River	Model	θ Lf	θ Lp	θ Ancestral population	M=4Nref*m Lp to Lf	M=4Nref*m from Lf to Lp	Tsplit/(4Nref)	Tsc/(4Nref)
		med [95HPD]	med [95HPD]	med [95HPD]	med [95HPD]	med [95HPD]	med [95HPD]	med [95HPD]
**AA**	IM	1.31[0.93.-2.02]	0.41[0.29-0.68]	2.29 [2.26-2.35.]	10 [7.0-12.6]	11.9 [10.9-12.8]	13.4 [12.3-14.1]	
	SC	1.48 [0.76.-2.55]	0.39 [0.27-0.62]	1.85 [1.60-2.11]	8.82 [8.10-9.98]	12.67 [11.8-13.25]	9.56 [8.40-11.5]	4.46 [3.07-5.40]
**BET**	IM	1.62 [1.26-2.35]	0.94 [0.43-1.54]	1.65 [1.50-1.78]	11.75 [10.96-12.72]	13.92 [13.02-14.93]	1.49 [1.21-1.71]	
	SC	1.93 [1.28-2.63]	0.94 [0.57-1.42]	1.16 [1.03-1.30]	9.44 [8.03-10.86]	13.28 [12.78-14.98]	16.14 [13.28-19.82]	8.23 [5.80-10.64]
**BRE**	IM	1.02 [0.44-2.45]	0.31 [0.12-0.79]	1.36 [0.44-2.37]	1.50 [0.39—6.06]	7.98 [2.43-15.49]	13.73 [4.99-22.69]	
	SC	1.61 [0.91-2.72]	0.74 [0.22-1.50]	1.44 [0.32-2.62]	6.68 [1.77-16.35]	9.88 [2.79-18.18]	13.48 [7.04-22.32]	1.02 [0.19.-6.23]
**HEM**	IM	1.00 [0.71-.194]	0.19 [0.13-0.31]	2.60 [2.48-2.70]	8.12 [6.73-10.75]	16.92 [15.74-17.68]	12.01 [9.56-13.50]	
	SC	0.86 [0.66-1.28]	0.35 [0.08-0.88]	1.68 [1.50-1.88]	12.45 [11.13-13.64]	9.67 [7.83-11.72]	13.93 [11.57-16.44]	4.97 [4.27-5.50]
**RIS**	IM	0.84 [0.60-1.54]	0.62 [0.50-0.88]	0.84 [0.77-0.93]	8.52 [5.90-11.96]	9.83 [7.24-12.55]	9.85 [9.09-10.47]	
	SC	1.36 [0.84-2.32]	0.64 [0.39-1.03]	1.01 [0.66-1.52]	14.45 [13.42-15.45]	14.78 [14.36-15.23]	11.31 [8.43-16.02]	4.56 [2.13-7.59]
**OIR**	PAN*	2.05 [1.94-2.18]						

Figure S1: Curves of out-of-bag errors rates and Estimation of variable importance for each population pairs.

Data based on one random forest, each composed of 1,000 trees obtained from a trained set of 50,000 simulated predictor variables (summary statistics). The response variable is the demographic model.

Each page corresponds to a different river:
(A) AA

(B) BET

(C) BRE

(D) HEM

(E) OIR

(F) RIS

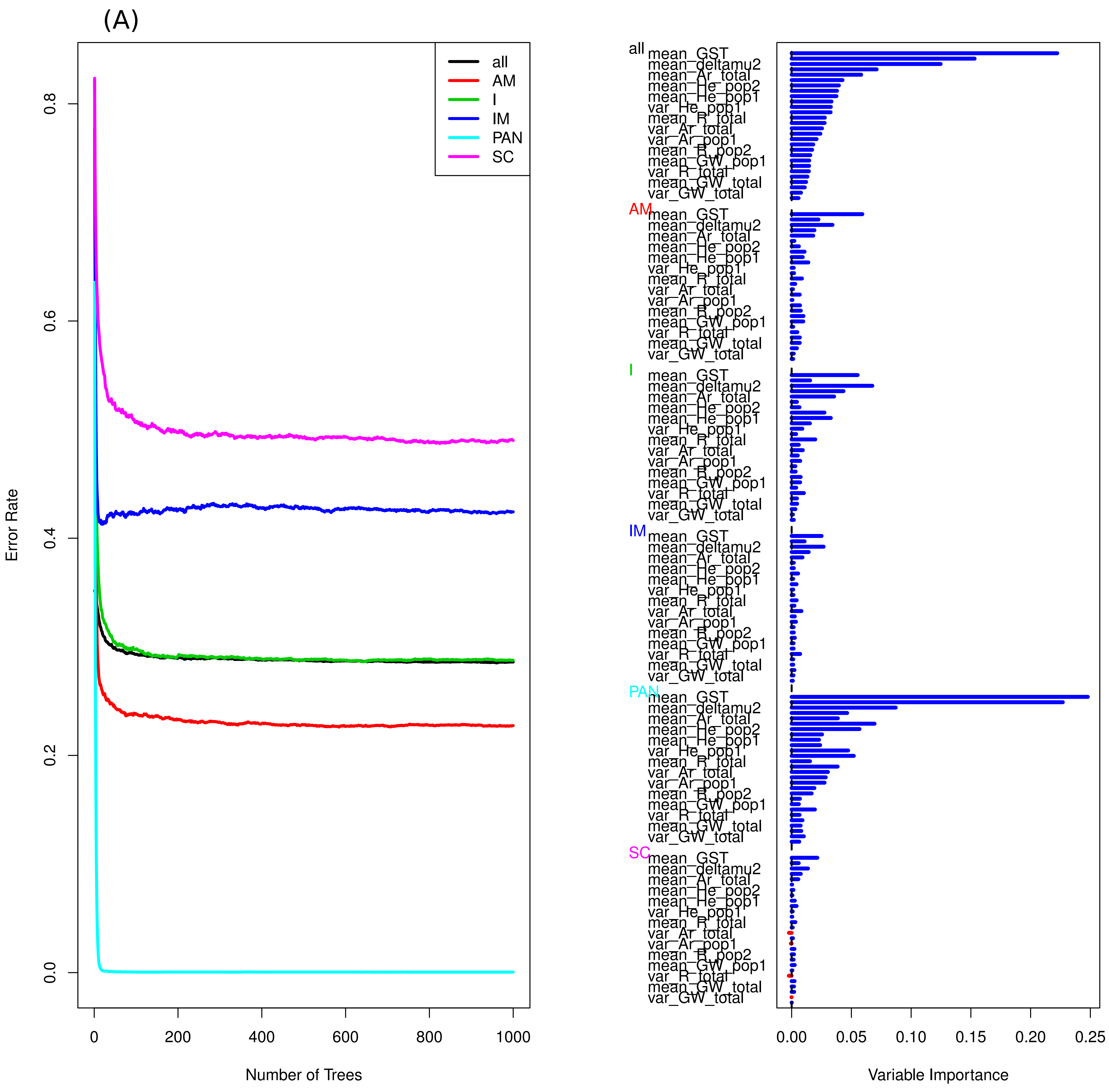

(A)
all
AM
I
IM
PAN
SC
Error Rate
0.0
0.2
0.4
0.6
0.8
0
200
400
600
800
1000
Number of Trees
all
mean_GST
mean_deltamu2
mean_Ar_total
mean_He_pop2
mean_He_pop1
var_He_pop1
mean_R_total
var_Ar_total
var_Ar_pop1
mean_R_pop2
mean_GW_pop1
var_R_total
mean_GW_total
var_GW_total
AM
mean_GST
mean_deltamu2
mean_Ar_total
mean_He_pop2
mean_He_pop1
var_He_pop1
mean_R_total
var_Ar_total
var_Ar_pop1
mean_R_pop2
mean_GW_pop1
var_R_total
mean_GW_total
var_GW_total
I
mean_GST
mean_deltamu2
mean_Ar_total
mean_He_pop2
mean_He_pop1
var_He_pop1
mean_R_total
var_Ar_total
var_Ar_pop1
mean_R_pop2
mean_GW_pop1
var_R_total
mean_GW_total
var_GW_total
IM
mean_GST
mean_deltamu2
mean_Ar_total
mean_He_pop2
mean_He_pop1
var_He_pop1
mean_R_total
var_Ar_total
var_Ar_pop1
mean_R_pop2
mean_GW_pop1
var_R_total
mean_GW_total
var_GW_total
PAN
mean_GST
mean_deltamu2
mean_Ar_total
mean_He_pop2
mean_He_pop1
var_He_pop1
mean_R_total
var_Ar_total
var_Ar_pop1
mean_R_pop2
mean_GW_pop1
var_R_total
mean_GW_total
var_GW_total
SC
mean_GST
mean_deltamu2
mean_Ar_total
mean_He_pop2
mean_He_pop1
var_He_pop1
mean_R_total
var_Ar_total
var_Ar_pop1
mean_R_pop2
mean_GW_pop1
var_R_total
mean_GW_total
var_GW_total
0.00
0.05
0.10
0.15
0.20
0.25
Variable Importance

(B)

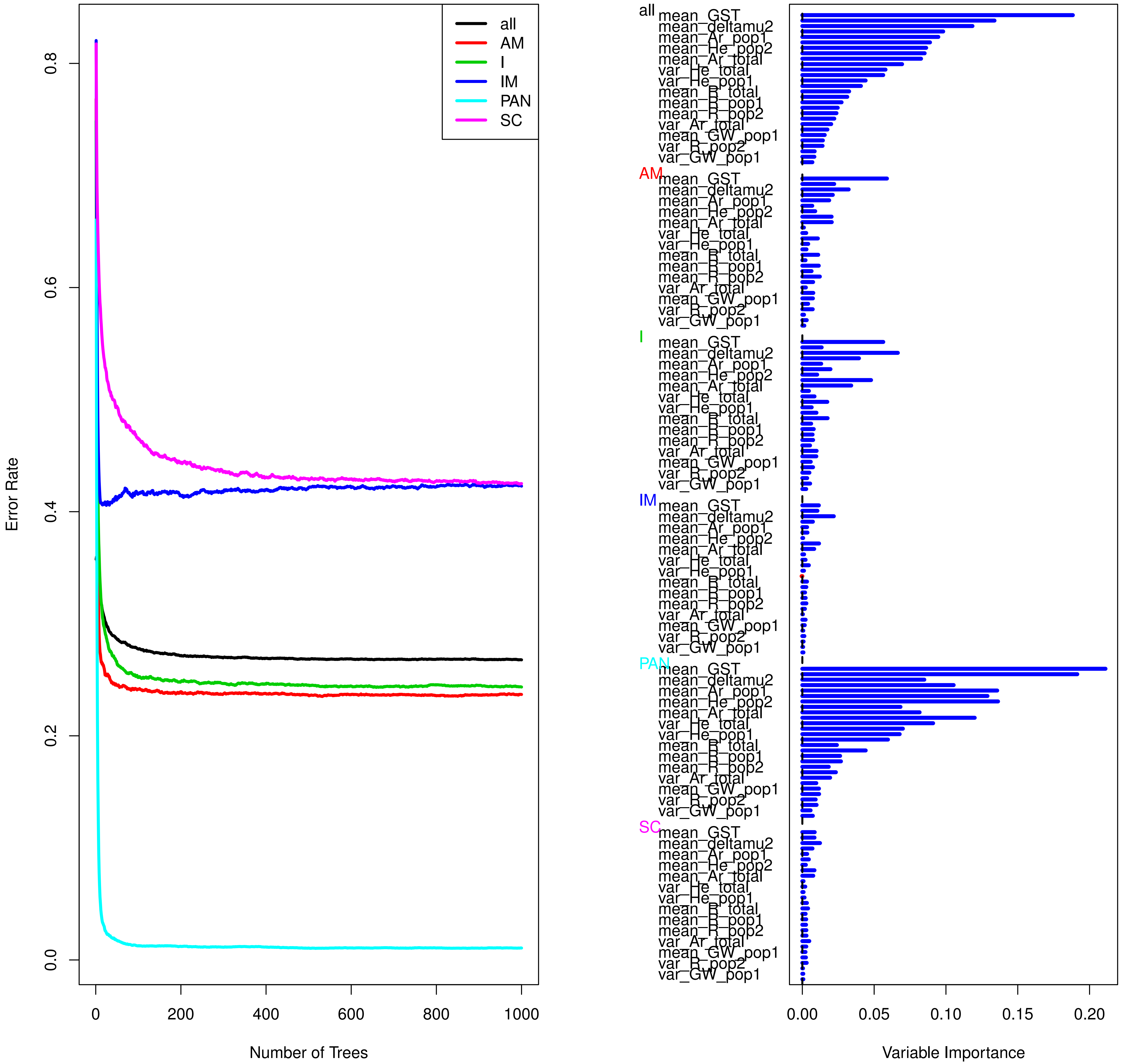

all
AM
I
IM
PAN
SC
Error Rate
0.0
0.2
0.4
0.6
0.8
0
200
400
600
800
1000
Number of Trees
mean_GST
mean_deltamu2
mean_Ar_pop1
mean_He_pop2
mean_Ar_total
var_He_total
var_He_pop1
mean_B_total
mean_R_pop1
mean_R_pop2
var_Ar_total
mean_GW_pop1
var_R_pop2
var_GW_pop1
0.00
0.05
0.10
0.15
0.20
Variable Importance

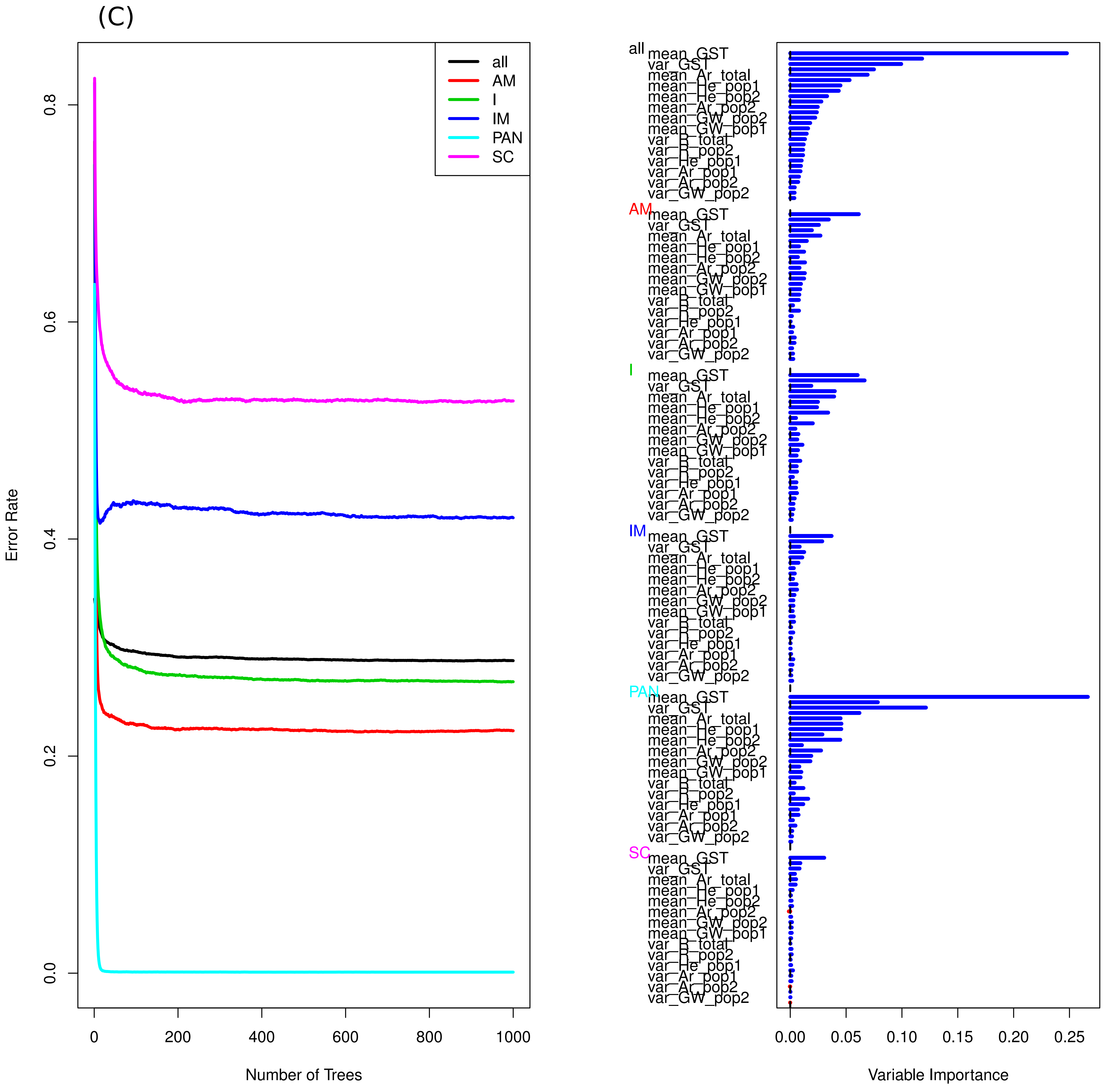

(C)
all
AM
I
IM
PAN
SC
Error Rate
Number of Trees
Variable Importance
mean_GST
var_GST
mean_Ar_total
mean_He_pop1
mean_He_pop2
mean_Ar_pop2
mean_GW_pop2
mean_GW_pop1
var_R_total
var_R_pop2
var_He_pop1
var_Ar_pop1
var_Ar_pop2
var_GW_pop2

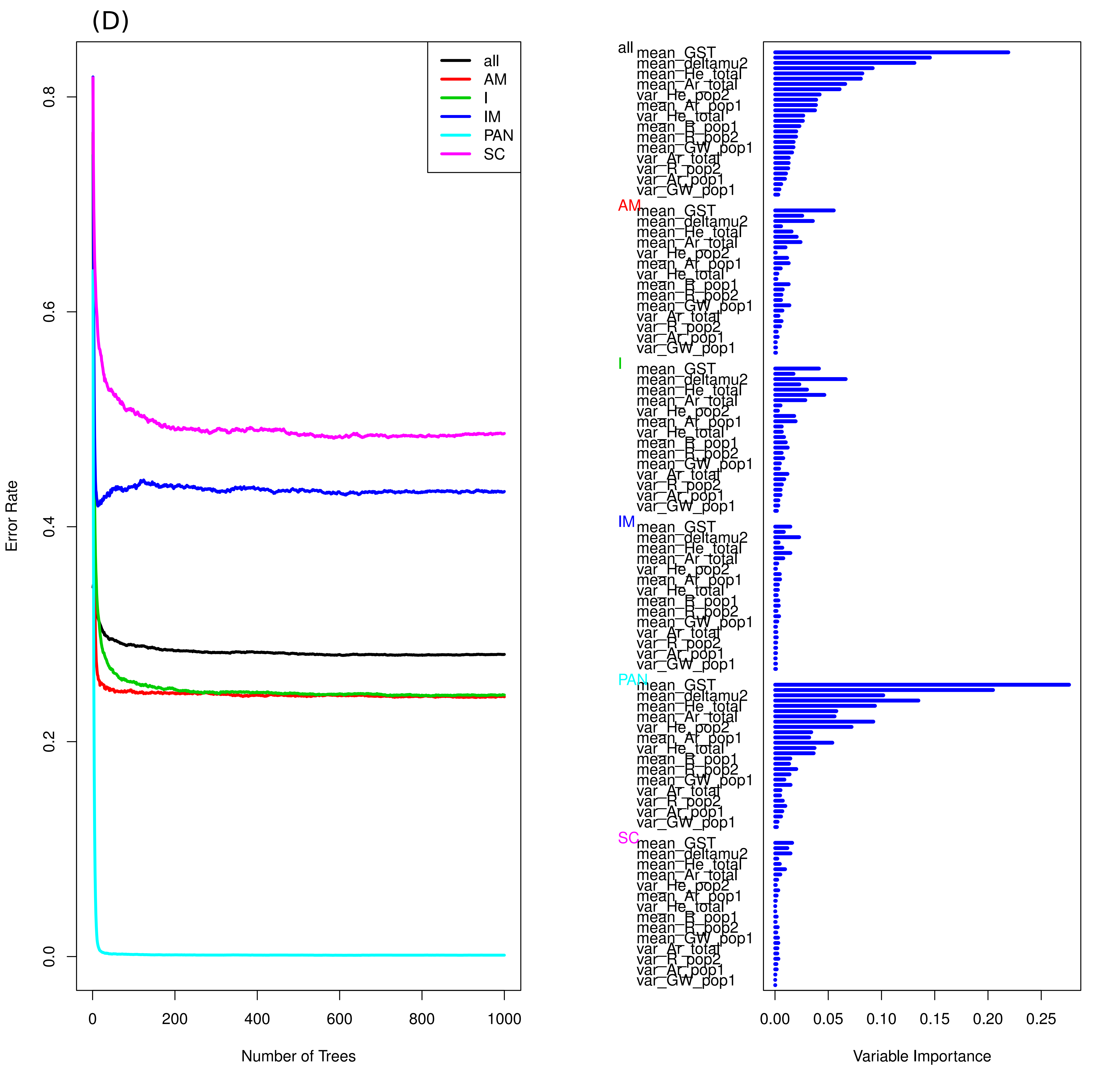

(D)
all
AM
I
IM
PAN
SC
Error Rate
0.0
0.2
0.4
0.6
0.8
0
200
400
600
800
1000
Number of Trees
all
mean_GST
mean_deltamu2
mean_He_total
mean_Ar_total
var_He_pop2
mean_Ar_pop1
var_He_total
mean_R_pop1
mean_R_pop2
mean_GW_pop1
var_Ar_total
var_R_pop2
var_Ar_pop1
var_GW_pop1
AM
I
IM
PAN
SC
0.00
0.05
0.10
0.15
0.20
0.25
Variable Importance

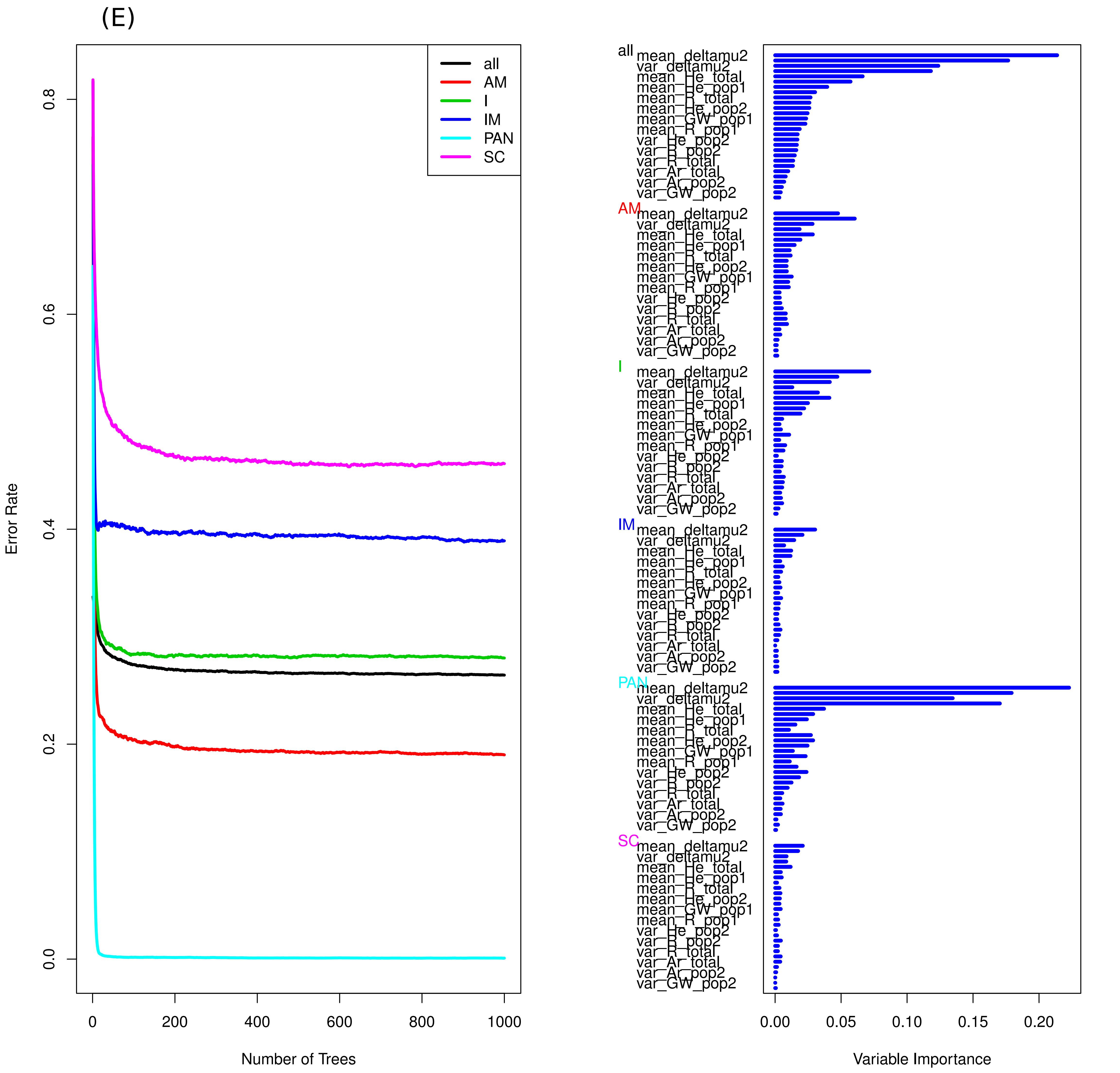

(E)
all
AM
I
IM
PAN
SC
Error Rate
0.0
0.2
0.4
0.6
0.8
0
200
400
600
800
1000
Number of Trees
mean_deltamu2
var_deltamu2
mean_He_total
mean_He_pop1
mean_R_total
mean_He_pop2
mean_GW_pop1
mean_R_pop1
var_He_pop2
var_R_pop2
var_R_total
var_Ar_total
var_Ar_pop2
var_GW_pop2
0.00
0.05
0.10
0.15
0.20
Variable Importance

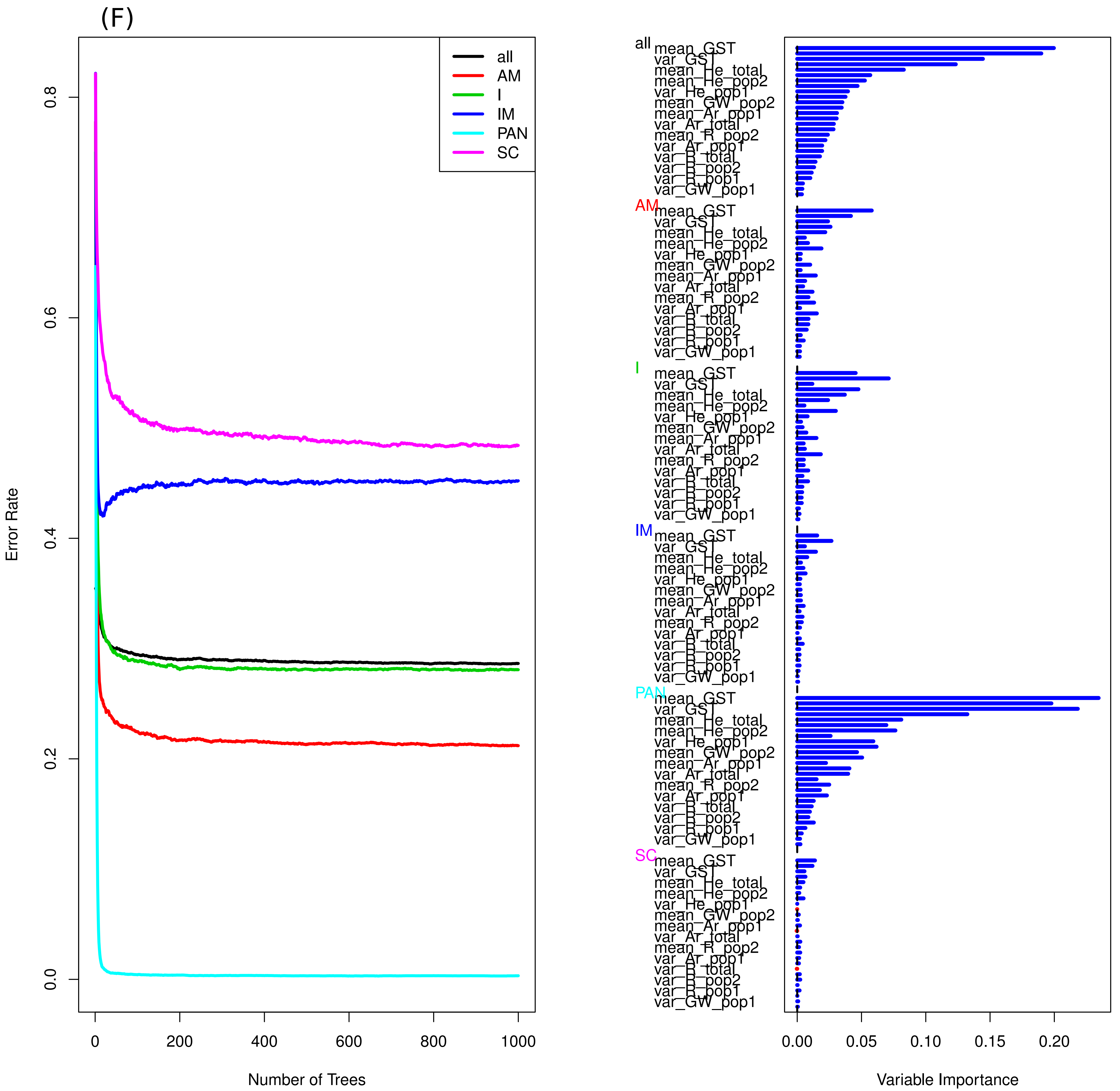
(F)
all
AM
I
IM
PAN
SC
Error Rate
Number of Trees
Variable Importance
mean_GST
var_GST
mean_He_total
mean_He_pop2
var_He_pop1
mean_GW_pop2
mean_Ar_pop1
var_Ar_total
mean_R_pop2
var_Ar_pop1
var_R_total
var_R_pop2
var_R_pop1
var_GW_pop1

Figure S2: Prior and Posterior Distribution of parameters estimations in each river under the SC and IM models

a) Parameter estimations under scenarios of divergence with gene flow (IM)

b) Parameter estimations under scenarios of secondary contact (SC)

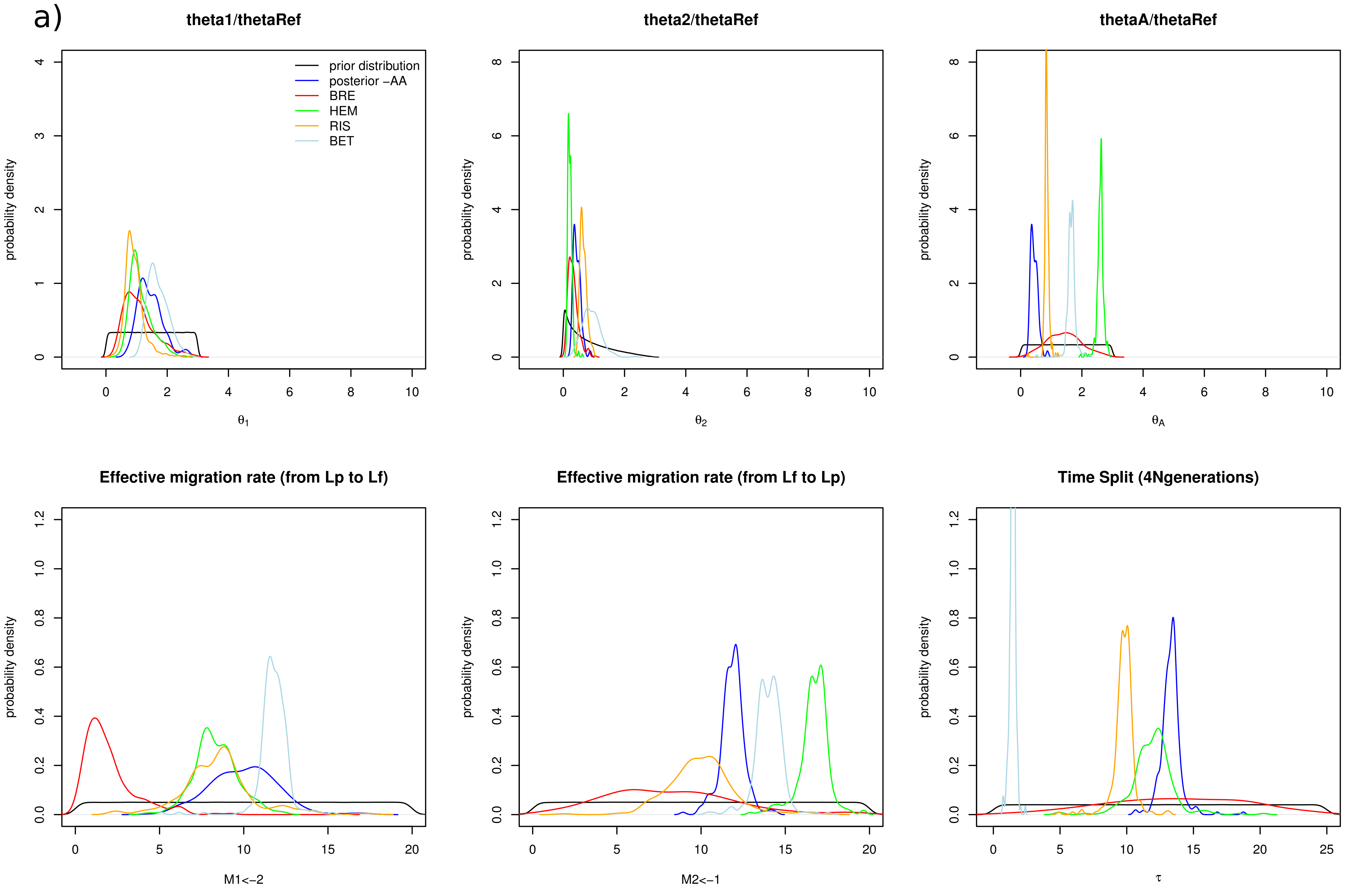

a)
theta1/thetaRef
theta2/thetaRef
thetaA/thetaRef
prior distribution
posterior −AA
BRE
HEM
RIS
BET
probability density
θ1
θ2
θA
Effective migration rate (from Lp to Lf)
Effective migration rate (from Lf to Lp)
Time Split (4Ngenerations)
M1<−2
M2<−1
τ

b)

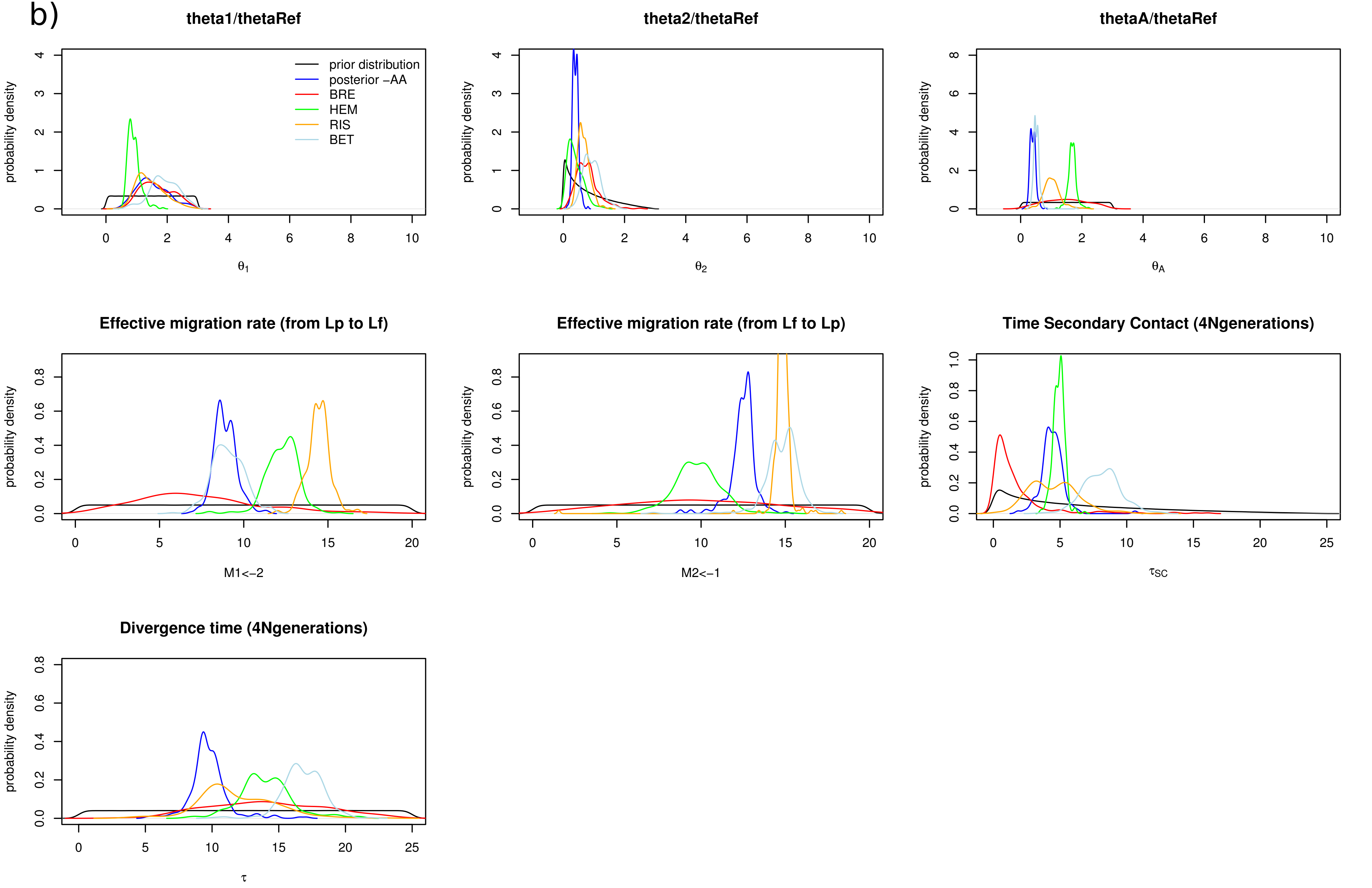

theta1/thetaRef
theta2/thetaRef
thetaA/thetaRef
prior distribution
posterior −AA
BRE
HEM
RIS
BET
probability density
θ1
θ2
θA
Effective migration rate (from Lp to Lf)
Effective migration rate (from Lf to Lp)
Time Secondary Contact (4Ngenerations)
M1<−2
M2<−1
τSC
Divergence time (4Ngenerations)
τ

Figure S2: Distribution of summary statistics obtained from the posterior predictive check.
Statistics obtained after 10 000 simulations based on posterior distributions.
Each page corresponds to a different river and a different model
(A) AA – IM

(B) BET – IM

(C) BRE – IM

(D) HEM – IM

(E) RIS – IM

(F) AA – SC

(G) BET – SC

(H) BRE – SC

(I) HEM – SC

(J) RIS – SC

(K) OIR – PAN

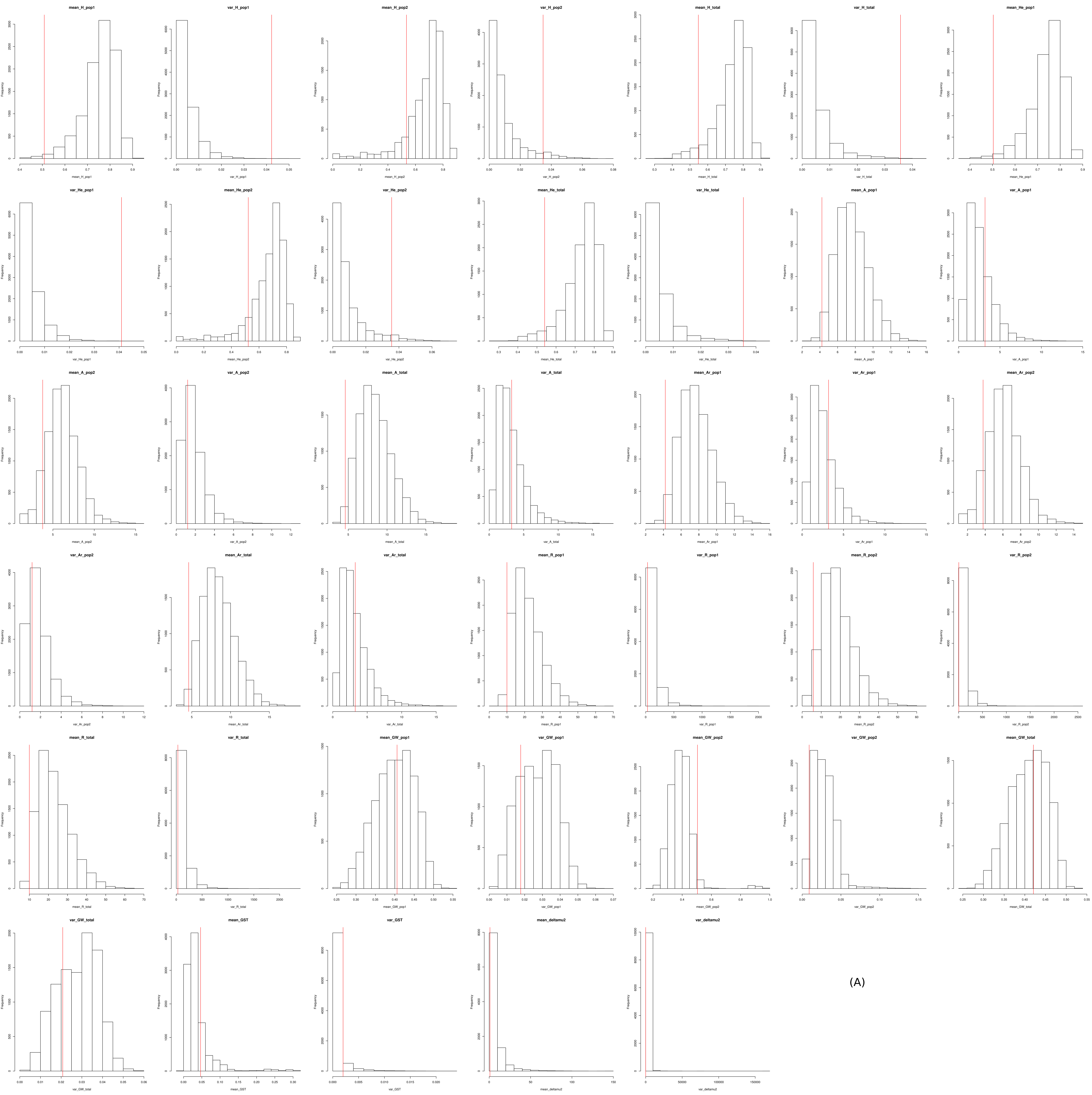
mean_H_pop1
var_H_pop1
mean_H_pop2
var_H_pop2
mean_H_total
var_H_total
mean_He_pop1
var_He_pop1
mean_He_pop2
var_He_pop2
mean_He_total
var_He_total
mean_A_pop1
var_A_pop1
mean_A_pop2
var_A_pop2
mean_A_total
var_A_total
mean_Ar_pop1
var_Ar_pop1
mean_Ar_pop2
var_Ar_pop2
mean_Ar_total
var_Ar_total
mean_R_pop1
var_R_pop1
mean_R_pop2
var_R_pop2
mean_R_total
var_R_total
mean_GW_pop1
var_GW_pop1
mean_GW_pop2
var_GW_pop2
mean_GW_total
var_GW_total
mean_GST
var_GST
mean_deltamu2
var_deltamu2
Frequency

(A)

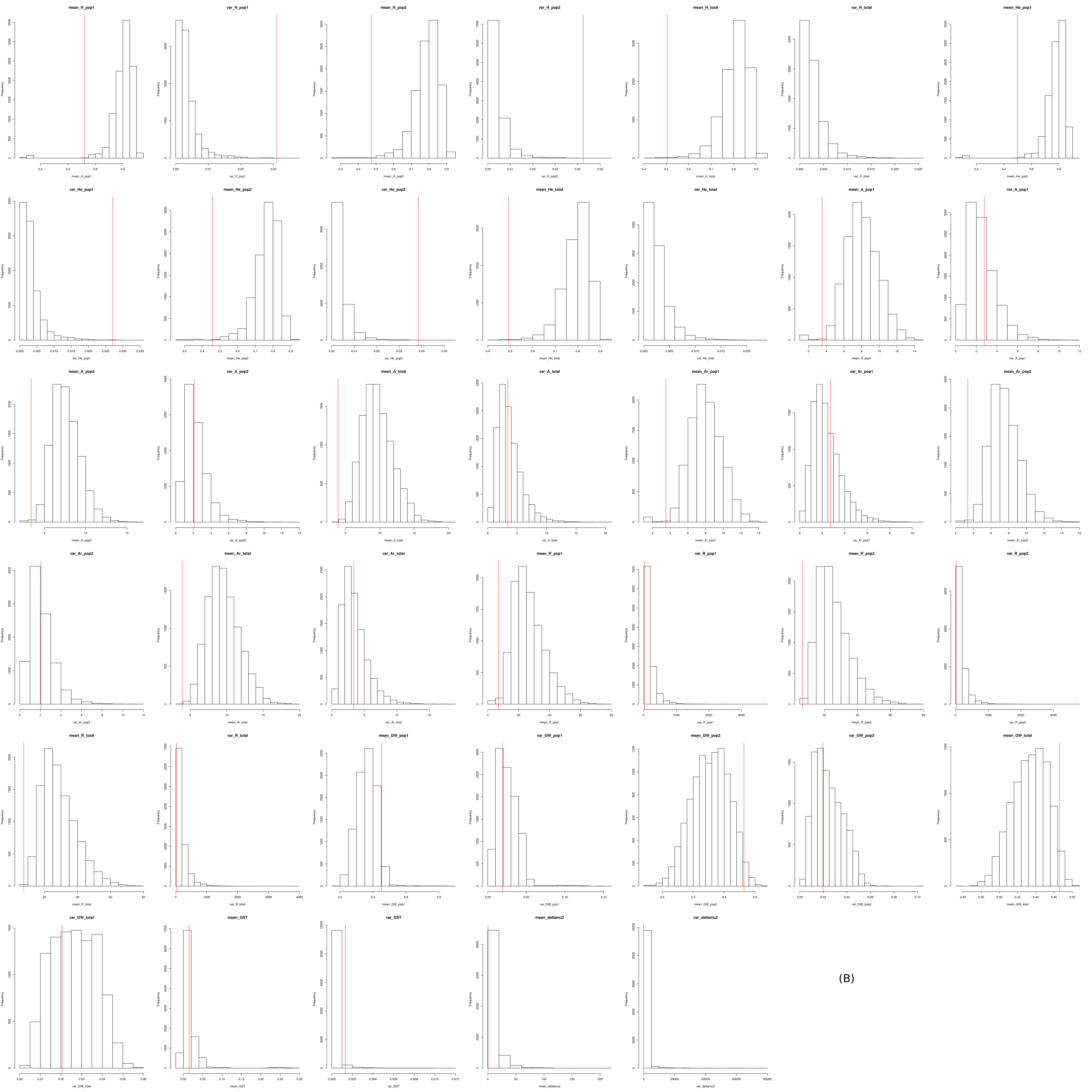
mean_H_pop1
var_H_pop1
mean_H_pop2
var_H_pop2
mean_H_total
var_H_total
mean_He_pop1
var_He_pop1
mean_He_pop2
var_He_pop2
mean_He_total
var_He_total
mean_A_pop1
var_A_pop1
mean_A_pop2
var_A_pop2
mean_A_total
var_A_total
mean_Ar_pop1
var_Ar_pop1
mean_Ar_pop2
var_Ar_pop2
mean_Ar_total
var_Ar_total
mean_R_pop1
var_R_pop1
mean_R_pop2
var_R_pop2
mean_R_total
var_R_total
mean_GW_pop1
var_GW_pop1
mean_GW_pop2
var_GW_pop2
mean_GW_total
var_GW_total
mean_GST
var_GST
mean_deltamu2
var_deltamu2
Frequency

(B)

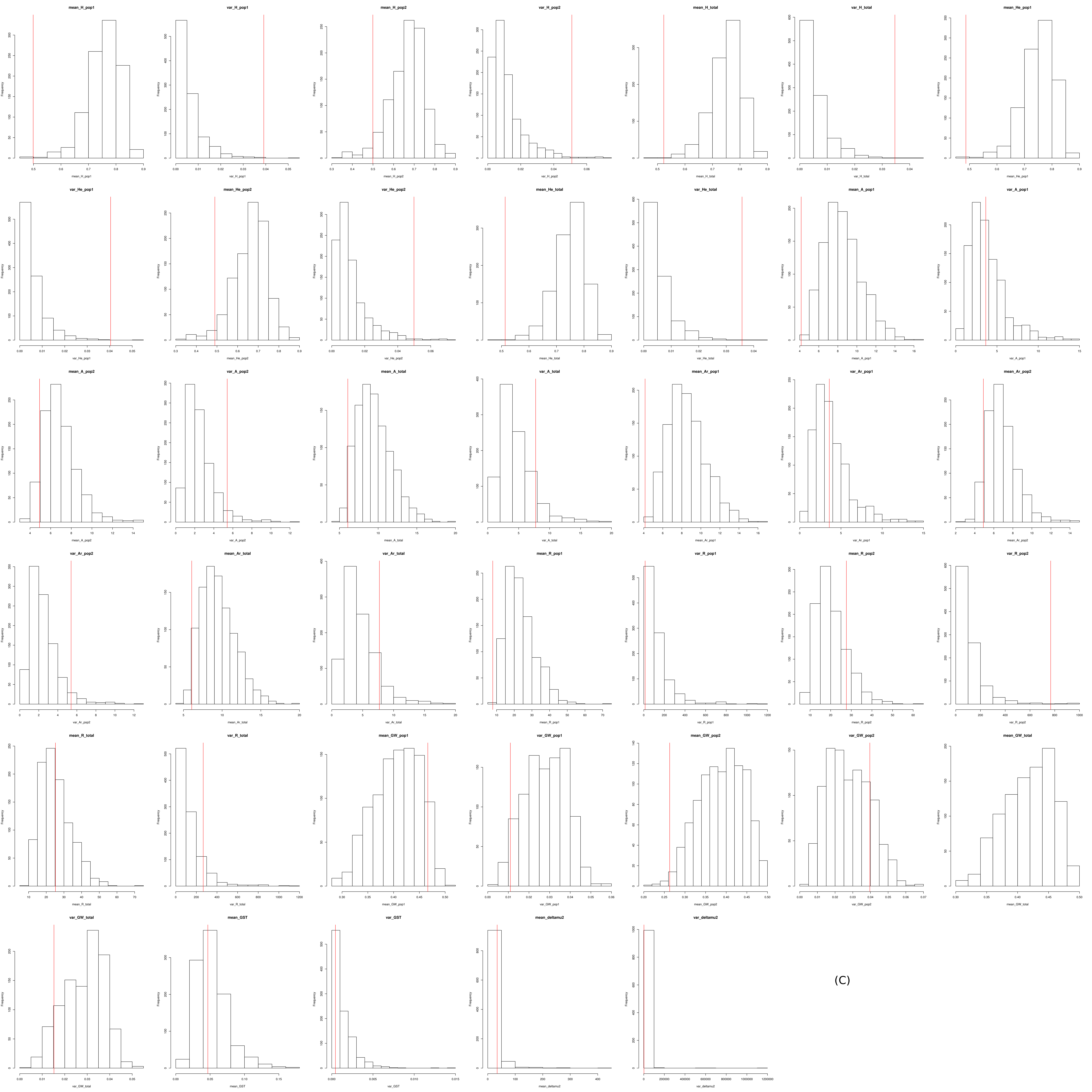

(C)

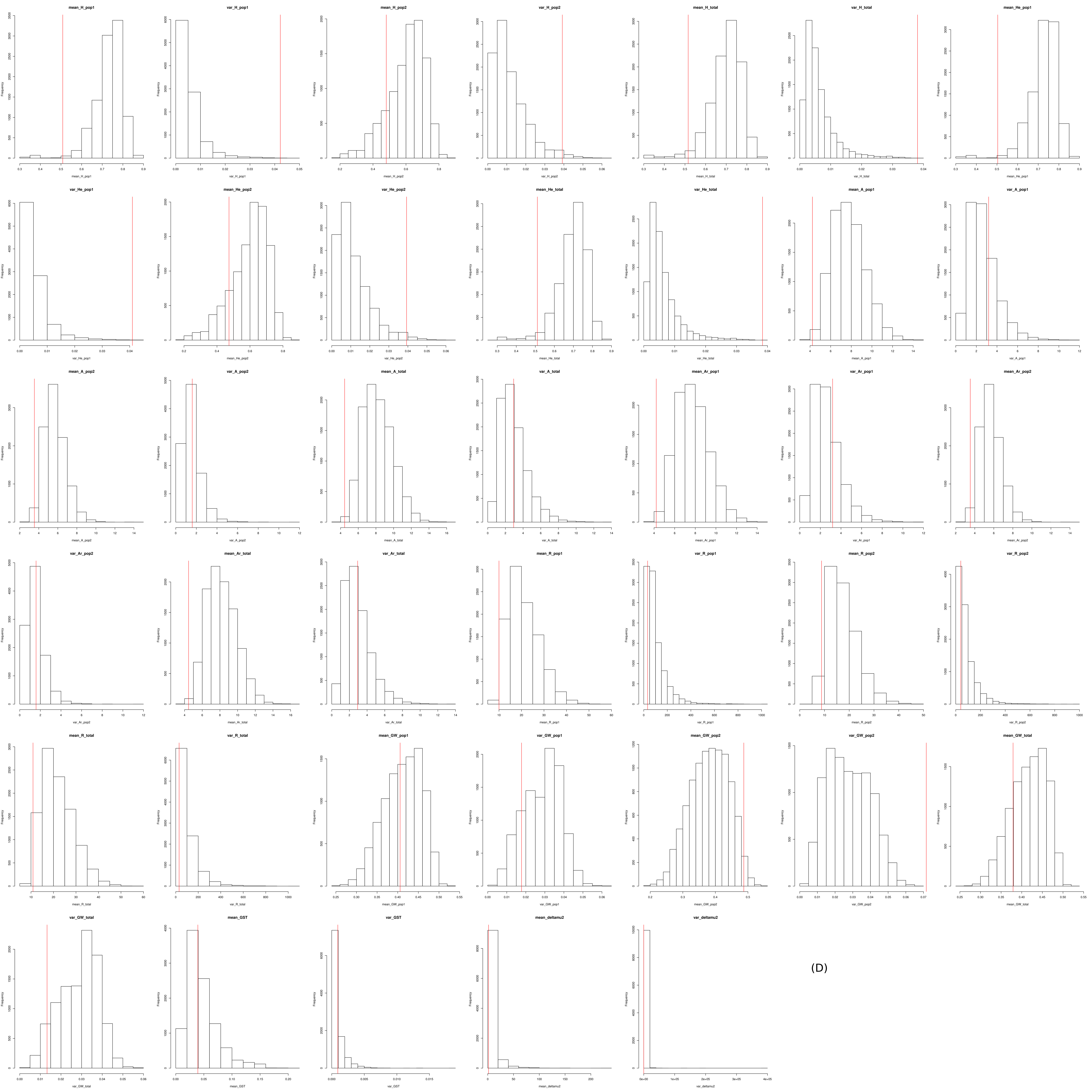

mean_H_pop1
var_H_pop1
mean_H_pop2
var_H_pop2
mean_H_total
var_H_total
mean_He_pop1
var_He_pop1
mean_He_pop2
var_He_pop2
mean_He_total
var_He_total
mean_A_pop1
var_A_pop1
mean_A_pop2
var_A_pop2
mean_A_total
var_A_total
mean_Ar_pop1
var_Ar_pop1
mean_Ar_pop2
var_Ar_pop2
mean_Ar_total
var_Ar_total
mean_R_pop1
var_R_pop1
mean_R_pop2
var_R_pop2
mean_R_total
var_R_total
mean_GW_pop1
var_GW_pop1
mean_GW_pop2
var_GW_pop2
mean_GW_total
var_GW_total
mean_GST
var_GST
mean_deltamu2
var_deltamu2

(D)

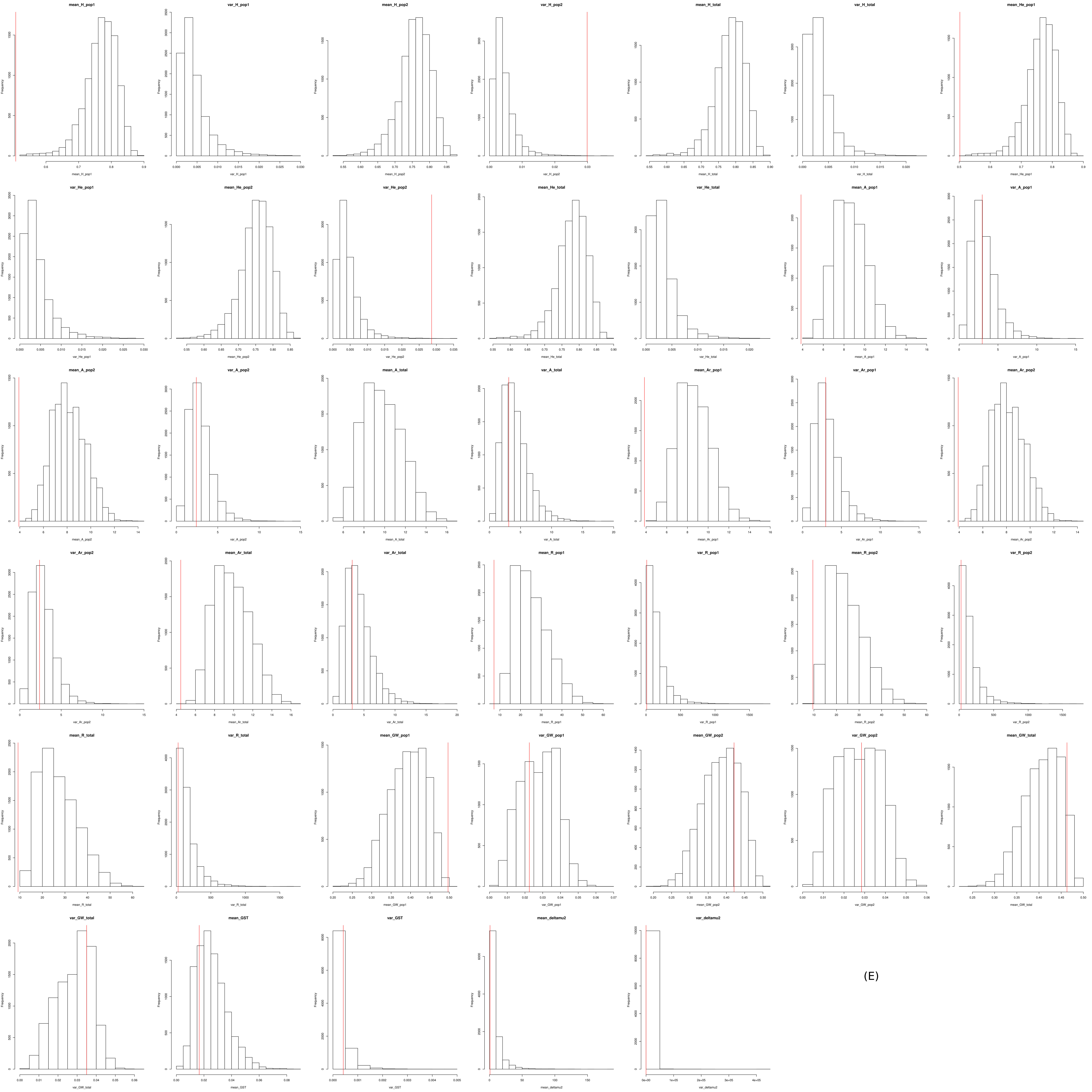
mean_H_pop1
var_H_pop1
mean_H_pop2
var_H_pop2
mean_H_total
var_H_total
mean_He_pop1
var_He_pop1
mean_He_pop2
var_He_pop2
mean_He_total
var_He_total
mean_A_pop1
var_A_pop1
mean_A_pop2
var_A_pop2
mean_A_total
var_A_total
mean_Ar_pop1
var_Ar_pop1
mean_Ar_pop2
var_Ar_pop2
mean_Ar_total
var_Ar_total
mean_R_pop1
var_R_pop1
mean_R_pop2
var_R_pop2
mean_R_total
var_R_total
mean_GW_pop1
var_GW_pop1
mean_GW_pop2
var_GW_pop2
mean_GW_total
var_GW_total
mean_GST
var_GST
mean_deltamu2
var_deltamu2
Frequency
(E)

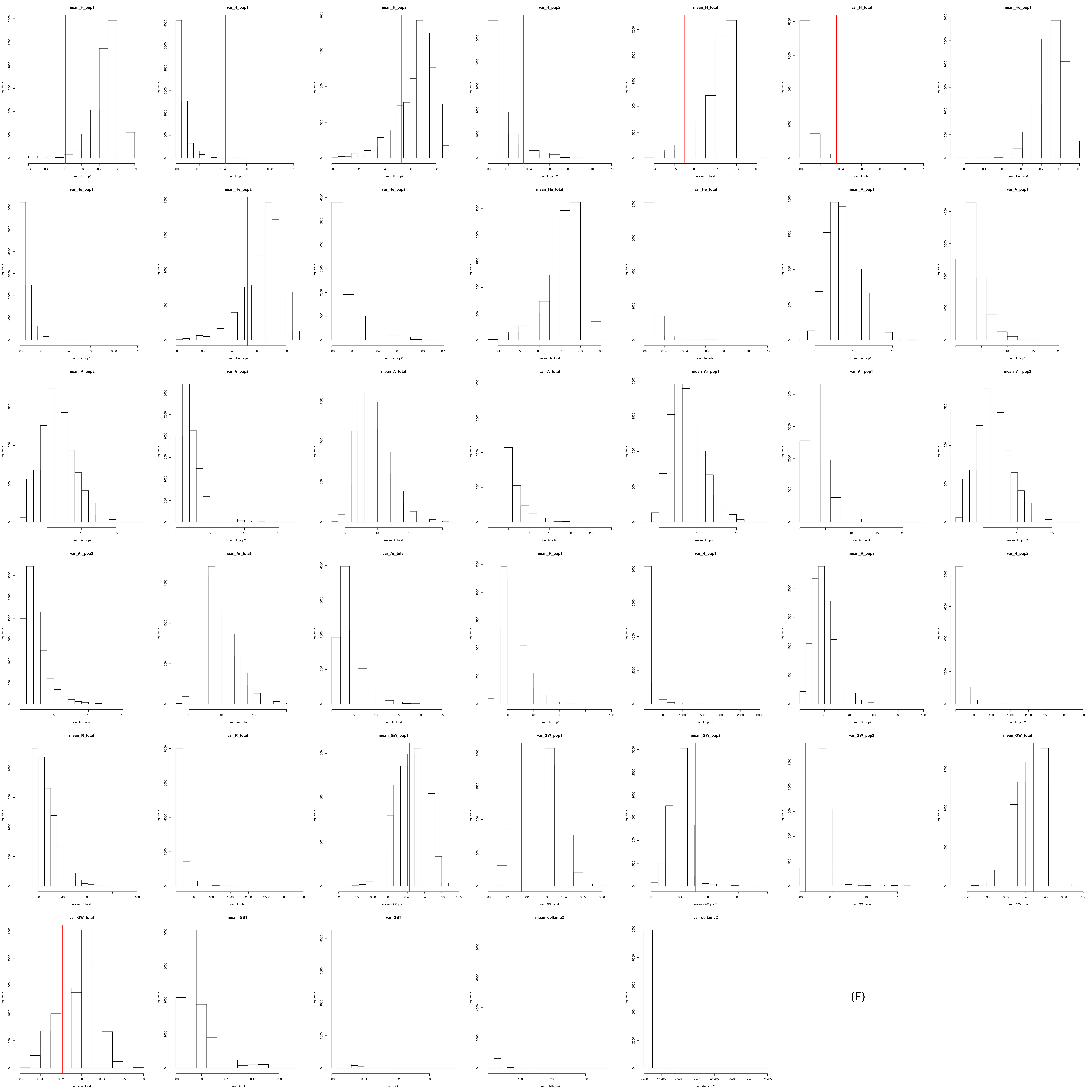
mean_H_pop1
var_H_pop1
mean_H_pop2
var_H_pop2
mean_H_total
var_H_total
mean_He_pop1
var_He_pop1
mean_He_pop2
var_He_pop2
mean_He_total
var_He_total
mean_A_pop1
var_A_pop1
mean_A_pop2
var_A_pop2
mean_A_total
var_A_total
mean_Ar_pop1
var_Ar_pop1
mean_Ar_pop2
var_Ar_pop2
mean_Ar_total
var_Ar_total
mean_R_pop1
var_R_pop1
mean_R_pop2
var_R_pop2
mean_R_total
var_R_total
mean_GW_pop1
var_GW_pop1
mean_GW_pop2
var_GW_pop2
mean_GW_total
var_GW_total
mean_GST
var_GST
mean_deltamu2
var_deltamu2
Frequency
(F)

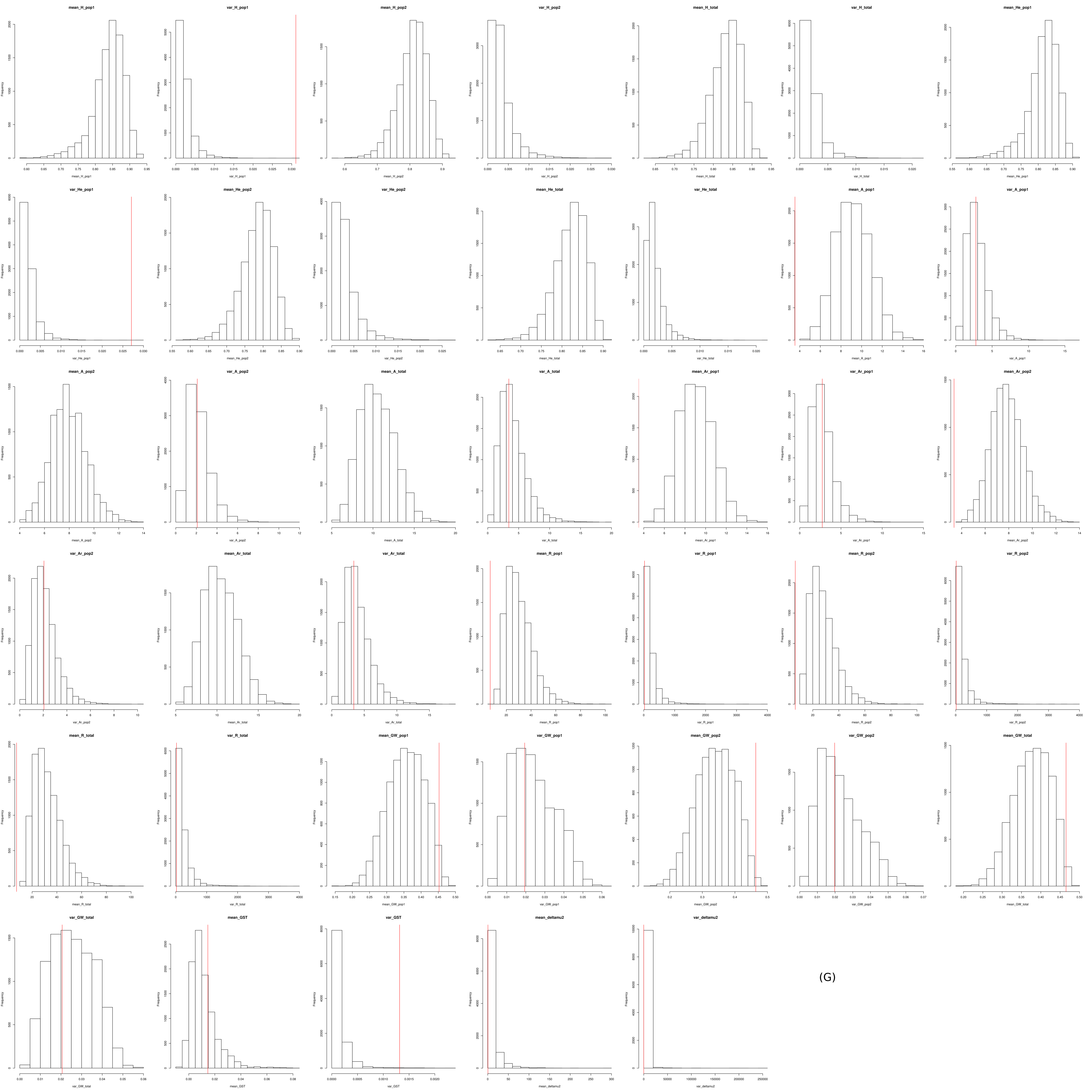
mean_H_pop1
var_H_pop1
mean_H_pop2
var_H_pop2
mean_H_total
var_H_total
mean_He_pop1
var_He_pop1
mean_He_pop2
var_He_pop2
mean_He_total
var_He_total
mean_A_pop1
var_A_pop1
mean_A_pop2
var_A_pop2
mean_A_total
var_A_total
mean_Ar_pop1
var_Ar_pop1
mean_Ar_pop2
var_Ar_pop2
mean_Ar_total
var_Ar_total
mean_R_pop1
var_R_pop1
mean_R_pop2
var_R_pop2
mean_R_total
var_R_total
mean_GW_pop1
var_GW_pop1
mean_GW_pop2
var_GW_pop2
mean_GW_total
var_GW_total
mean_GST
var_GST
mean_deltamu2
var_deltamu2
Frequency

(G)

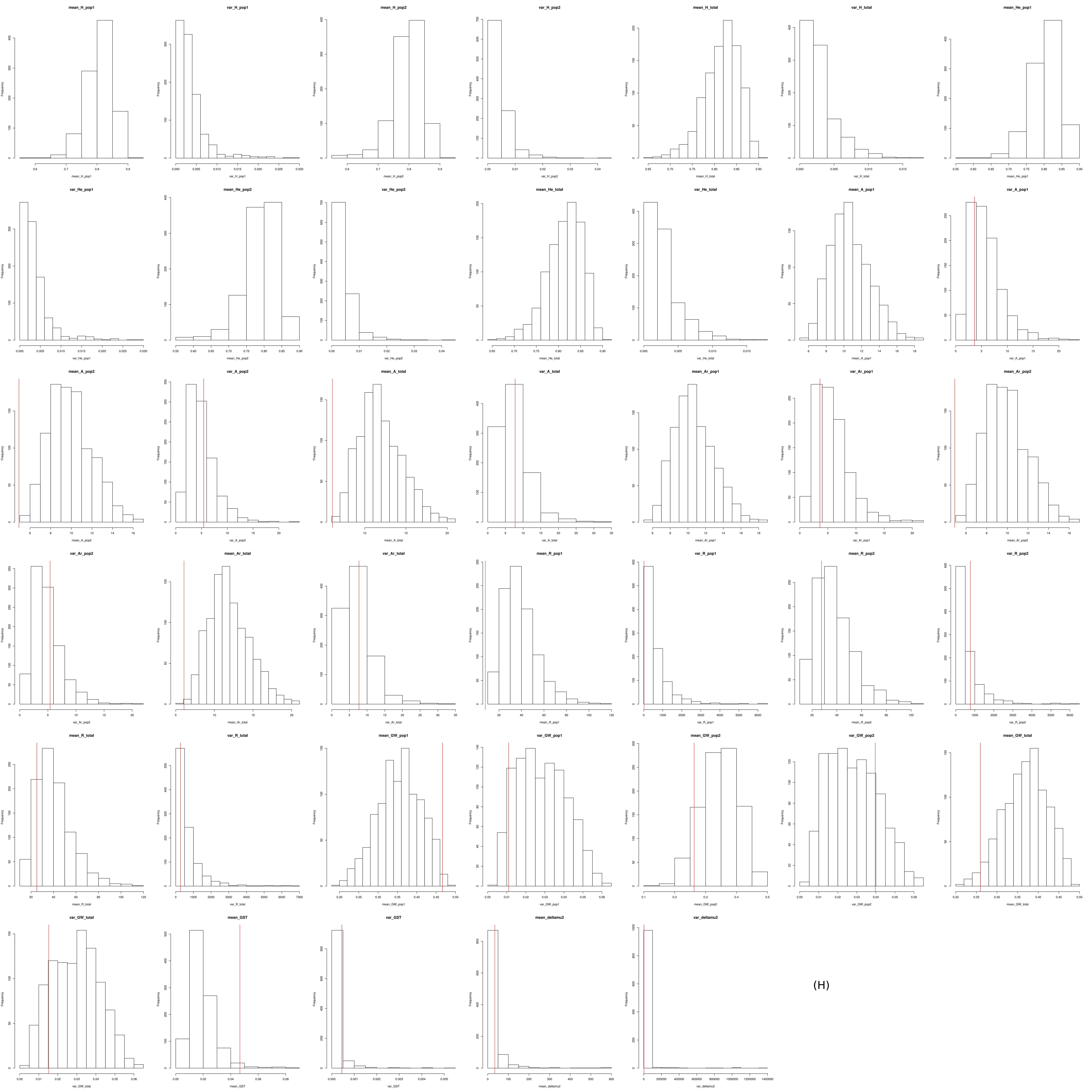
mean_H_pop1
var_H_pop1
mean_H_pop2
var_H_pop2
mean_H_total
var_H_total
mean_He_pop1
var_He_pop1
mean_He_pop2
var_He_pop2
mean_He_total
var_He_total
mean_A_pop1
var_A_pop1
mean_A_pop2
var_A_pop2
mean_A_total
var_A_total
mean_Ar_pop1
var_Ar_pop1
mean_Ar_pop2
var_Ar_pop2
mean_Ar_total
var_Ar_total
mean_R_pop1
var_R_pop1
mean_R_pop2
var_R_pop2
mean_R_total
var_R_total
mean_GW_pop1
var_GW_pop1
mean_GW_pop2
var_GW_pop2
mean_GW_total
var_GW_total
mean_GST
var_GST
mean_deltamu2
var_deltamu2
Frequency

(H)

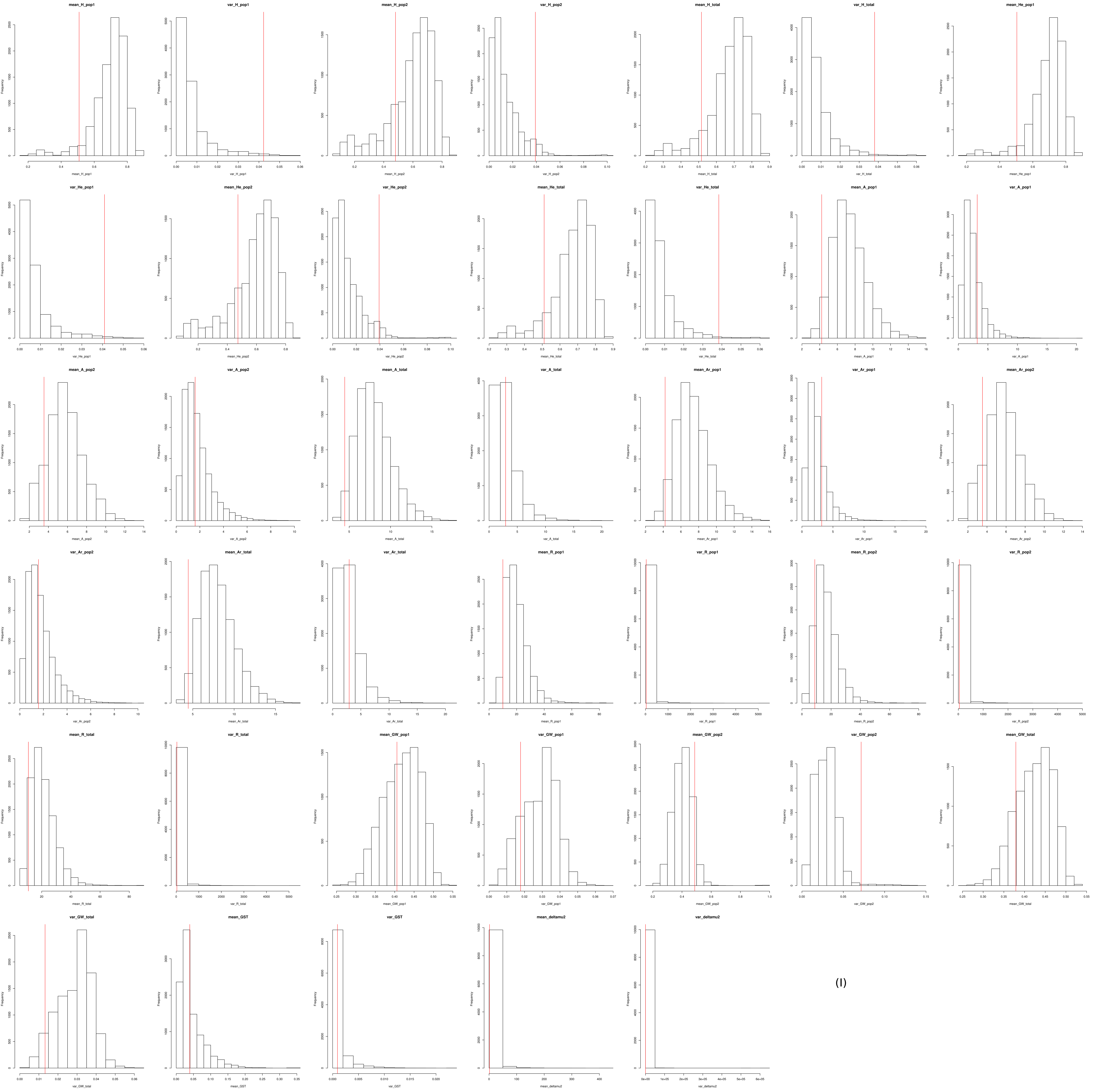
mean_H_pop1
var_H_pop1
mean_H_pop2
var_H_pop2
mean_H_total
var_H_total
mean_He_pop1
var_He_pop1
mean_He_pop2
var_He_pop2
mean_He_total
var_He_total
mean_A_pop1
var_A_pop1
mean_A_pop2
var_A_pop2
mean_A_total
var_A_total
mean_Ar_pop1
var_Ar_pop1
mean_Ar_pop2
var_Ar_pop2
mean_Ar_total
var_Ar_total
mean_R_pop1
var_R_pop1
mean_R_pop2
var_R_pop2
mean_R_total
var_R_total
mean_GW_pop1
var_GW_pop1
mean_GW_pop2
var_GW_pop2
mean_GW_total
var_GW_total
mean_GST
var_GST
mean_deltamu2
var_deltamu2
Frequency

(I)

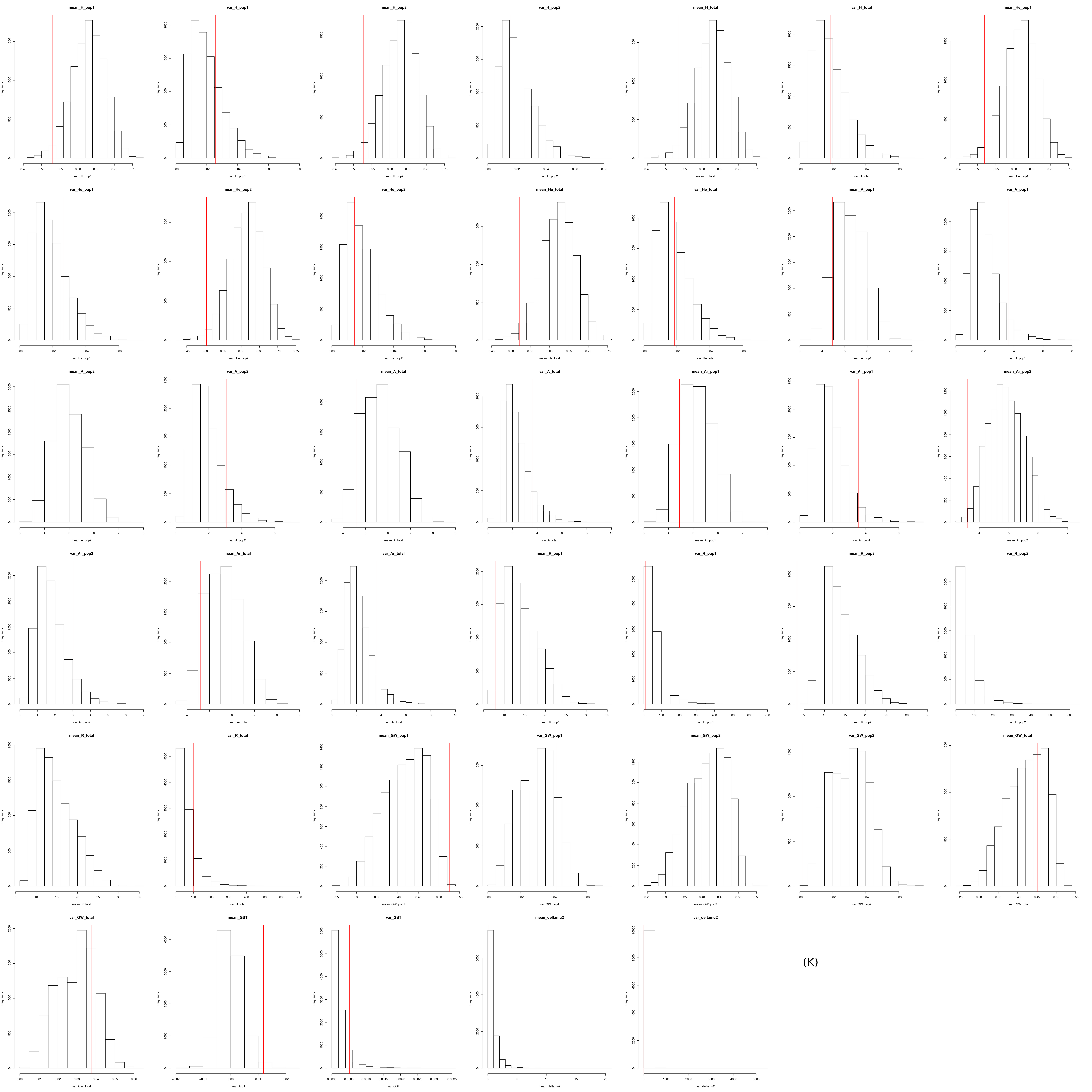
mean_H_pop1
var_H_pop1
mean_H_pop2
var_H_pop2
mean_H_total
var_H_total
mean_He_pop1
var_He_pop1
mean_He_pop2
var_He_pop2
mean_He_total
var_He_total
mean_A_pop1
var_A_pop1
mean_A_pop2
var_A_pop2
mean_A_total
var_A_total
mean_Ar_pop1
var_Ar_pop1
mean_Ar_pop2
var_Ar_pop2
mean_Ar_total
var_Ar_total
mean_R_pop1
var_R_pop1
mean_R_pop2
var_R_pop2
mean_R_total
var_R_total
mean_GW_pop1
var_GW_pop1
mean_GW_pop2
var_GW_pop2
mean_GW_total
var_GW_total
mean_GST
var_GST
mean_deltamu2
var_deltamu2

(K)
